# First experiences with dynamic renal [^68^Ga]Ga-DOTA PET/CT: a comparison to renal scintigraphy and compartmental modelling to non-invasively estimate the glomerular filtration rate

**DOI:** 10.1007/s00259-022-05781-1

**Published:** 2022-04-12

**Authors:** David Kersting, Miriam Sraieb, Robert Seifert, Pedro Fragoso Costa, Sandra Kazek, Lukas Kessler, Lale Umutlu, Wolfgang Peter Fendler, Walter Jentzen, Ken Herrmann, Florian Büther, Michael Nader, Christoph Rischpler

**Affiliations:** 1 Department of Nuclear Medicine, University Hospital Essen, West German Cancer Center (WTZ), University of Duisburg-Essen, Hufelandstrasse 55, 45147 Essen, Germany; 2 German Cancer Consortium (DKTK, Partner Site Essen/Düsseldorf), Essen, Germany; 3 Department of Nuclear Medicine, University Hospital Muenster, University of Muenster, Muenster, Germany; 4 Department of Diagnostic and Interventional Radiology and Neuroradiology, University Hospital Essen, University of Duisburg-Essen, Essen, Germany

**Keywords:** DOTA PET, Renal PET/CT, Glomerular filtration rate, Compartmental kinetic modelling, Dynamic PET

## Abstract

**Purpose:**

The determination of the glomerular filtration rate (GFR) is decisive for a variety of clinical issues, for example, to monitor the renal function in radionuclide therapy patients. Renal scintigraphy using glomerularly filtered tracers allows combined acquisition of renograms and GFR estimation but requires repeated blood sampling for several hours. In contrast, dynamic PET imaging using the glomerularly filtered tracer [^68^Ga]Ga-DOTA bears the potential to non-invasively estimate the GFR by compartmental kinetic modelling. Here, we report the, to our knowledge, first comparison of human renal dynamic [^68^Ga]Ga-DOTA PET imaging in comparison to renal scintigraphy and compare PET-derived to serum creatinine-derived GFR measurements.

**Methods:**

Dynamic [^68^Ga]Ga-DOTA PET data were acquired for 30 min immediately after tracer injection in 12 patients. PET and renal scintigraphy images were visually interpreted in a consensus read by three nuclear medicine physicians. The functional renal cortex was segmented to obtain time-activity curves. The arterial input function was estimated from the PET signal in the abdominal aorta. Single-compartmental tracer kinetic modelling was performed to calculate the GFR using complete 30-min (GFR_PET-30_) and reduced 15-min PET data sets (GFR_PET-15_) to evaluate whether a shorter acquisition time is sufficient for an accurate GFR estimation. A modified approach excluding minutes 2 to 10 was applied to reduce urinary spill-over effects. Serum creatinine-derived GFR_CKD_ (CKD-EPI-formula) was used as reference standard.

**Results:**

PET image interpretation revealed the same findings as conventional scintigraphy (2/12 patients with both- and 1/12 patients with right-sided urinary obstruction). Model fit functions were substantially improved for the modified approach to exclude spill-over. Depending on the modelling approach, GFR_CKD_ and both GFR_PET-30_ and GFR_PET-15_ were well correlated with interclass correlation coefficients (ICCs) from 0.74 to 0.80 and Pearson’s correlation coefficients (PCCs) from 0.74 to 0.81. For a subgroup of patients with undisturbed urinary efflux (*n* = 9), correlations were good to excellent (ICCs from 0.82 to 0.95 and PCCs from 0.83 to 0.95). Overall, GFR_PET-30_ and GFR_PET-15_ were excellently correlated (ICCs from 0.96 to 0.99 and PCCs from 0.96 to 0.99).

**Conclusion:**

Renal [^68^Ga]Ga-DOTA PET can be a suitable alternative to conventional scintigraphy. Visual assessment of PET images and conventional renograms revealed comparable results. GFR values derived by non-invasive single-compartmental-modelling of PET data show a good correlation to serum creatinine-derived GFR values. In patients with undisturbed urinary efflux, the correlation was excellent. Dynamic PET data acquisition for 15 min is sufficient for visual evaluation and GFR derivation.

**Supplementary Information:**

The online version contains supplementary material available at 10.1007/s00259-022-05781-1.

## Introduction

The glomerular filtration rate (GFR) is the standard metric of renal function in clinical routine and decisive for a variety of clinical issues. For example, staging of chronic kidney diseases [1] and drug dose adjustment in kidney disease patients [2] are based on GFR measurements. Prior to organ transplantation, GFR estimates are performed in living kidney donors to analyse the renal function [3]. In nuclear medicine, the GFR is used to ensure an adequate kidney function in radionuclide therapy patients [4, 5].

As the GFR cannot directly be measured, several indirect methods are established in routine clinical practice [6]. These include urinary or plasma clearance measurements of endogenous and exogeneous filtration markers or estimations based on serum measurements of endogenous filtration markers [7]. Mostly, the GFR is derived from serum creatinine or serum cystatine C [6]. Various established equations can be used for a serum creatinine-derived estimation with the Chronic Kidney Disease—Epidemiology Collaboration (CKD-EPI) equation yielding most accurate results, particularly in individuals with higher GFR rates [8, 9]. The clinical gold standard of GFR measurement by urinary inulin clearance is, in contrast, not routinely performed due to the laborious procedure requiring continuous inulin injection and urine collection and the limited availability of the substance [2].

Renography in planar scintigraphy technique using radioactively-labelled markers like [^51^Cr]Cr-ethylenediamine-tetraacetic acid ([^51^Cr]Cr-EDTA), [^99m^Tc]Tc-diethylenetriamine-pentaacetic acid ([^99m^Tc]Tc-DTPA), or [^99m^Tc]Tc-mercaptoacetyltriglycine ([^99m^Tc]Tc-MAG3) is clinically well established to evaluate renal perfusion, functional uptake, cortical transit, and urinary excretion [10]. Moreover, for the renal scintigraphy tracers [^51^Cr]Cr-EDTA and [^99m^Tc]Tc-DTPA, which are in good agreement with the criteria of an ideal exogenous filtration marker to be freely filtered, not protein bound, not tubularly reabsorbed or secreted, and not renally metabolized [6], simultaneous renography and estimation of the GFR is possible [11]. However, repeated blood sampling over a period of several hours is required for an accurate GFR estimation [11, 12].

Alternatively, dynamic renal PET imaging can be performed using glomerularly filtered PET tracers like [^68^Ga]Ga-1,4,7-triaza-cyclononane-1,4,7-triacetic acid ([^68^Ga]Ga-NOTA), [^68^Ga]Ga-1,4,7,10-tetraaza-cyclododecane-1,4,7,10-tetraacetic acid ([^68^Ga]Ga-DOTA), or [^68^Ga]Ga-EDTA. On the one hand, advantages of imaging in PET technique are a higher spatial and temporal resolution, higher sensitivity, absolute quantification, and 3-dimensional (3D) imaging [13] resulting in an improved visualization of the renal parenchyma. On the other hand, dynamic renal PET bears the potential to estimate the GFR from PET images without venous blood sampling depending on the applied PET tracer [14–16]. For example, [^68^Ga]Ga-DOTA may be well suited as tracer for a PET-derived GFR estimation, as DOTA, which is used as Gd-DOTA in MR contrast agents, exhibits similar pharmacokinetics to DTPA [17, 18] and is almost exclusively cleared from the blood by glomerular filtration [19, 20]. A sophisticated method for PET-derived GFR estimation is compartmental kinetic modelling. Promising results for this technique were recently described for [^68^Ga]Ga-NOTA, a similar tracer to [^68^Ga]Ga-DOTA, in rats [14], but, to the best of our knowledge, it has not yet been evaluated in humans.

In our institution, PSMA- and DOTATOC-/DOTATATE-radionuclide therapy patients routinely undergo renography to monitor renal function, mostly in [^99m^Tc]Tc-MAG3 or [^99m^Tc]Tc-DTPA scintigraphy technique. We recently started to, alternatively, perform dynamic [^68^Ga]Ga-DOTA PET/CT imaging. We here report the, to our knowledge, first evaluation of human renal [^68^Ga]Ga-DOTA PET/CT imaging in comparison to renal scintigraphy. Additionally, we performed a PET-derived GFR estimation by single-compartmental tracer kinetic modelling to translate the method that was previously described in rats to human data. The results are compared to serum creatinine-derived measurements.

## Materials and methods

### Patient characteristics

Patient data sets of 10 males and 2 females radionuclide therapy patients (7 [^177^Lu]Lu-DOTATOC and 5 [^177^Lu]Lu-PSMA therapy patients, mean age 67 years) who underwent [^68^Ga]Ga-DOTA PET imaging were included. Detailed patient characteristics and blood test results are given in Table 1. The local institutional ethics committee (University of Duisburg-Essen, medical faculty) approved the study (Ethics protocol number 20–9594-BO).

**Table 1:** Patient characteristics including administered activities for PET and scintigraphy imaging and blood test results

Patient ID	Sex	Age	Body surface area (m^2^)	Radionuclide therapy	Serum creatinine (mg/dl)	HCT	Activity PET (MBq)	Scintigraphy tracer	Activity scintigraphy (MBq)
1	f	53	1.88	DOTATOC	0.89	0.39	137	MAG_3_	75
2	m	72	2.08	DOTATOC	1.41	0.32	109	DTPA	148
3	f	58	1.56	DOTATOC	0.60	0.31	112	MAG_3_	74
4	m	78	1.91	DOTATOC	0.85	0.40	102	DTPA	141
5	m	67	1.98	DOTATOC	0.99	0.39	79	DTPA	155
6	m	67	2.18	DOTATOC	1.26	0.35	94	DTPA	152
7	m	70	1.81	PSMA	0.92	0.35	112	DTPA	130
8	m	46	2.25	DOTATOC	0.90	0.40	120	MAG_3_	66
9	m	65	1.98	PSMA	0.88	0.26	113	DTPA	135
10	m	79	1.95	PSMA	1.15	0.38	120	MAG_3_	69
11	m	72	1.77	PSMA	0.66	0.29	119	DTPA	159
12	m	79	1.96	PSMA	0.95	0.29	125	DTPA	136

*HCT:* *haematocrit*

### [^68^Ga]Ga-DOTA preparation

^68^Ga was obtained from a 1100-MBq ^68^Ge/^68^Ga generator (Eckert & Ziegler Strahlen- und Medizintechnik AG, Berlin, Germany) by elution with 5 ml 0.1 M HCl solution. Labelling was performed using a Modular-Lab Eazy synthesizer (Eckert & Ziegler Strahlen- und Medizintechnik AG, Berlin, Germany). Before the automated synthesis started, the reaction vial was pre-loaded with 15 µg DOTA (Merck, Darmstadt) dissolved in 0.6 ml of sodium acetate buffer solution. The elution of the generators with 0.1 M HCl solution was performed fully automated. The solution was passed through a cation exchange cartridge (type PS-H^+^). ^68^Ga was eluted from the PS-H+ cartridge into the reaction vial using eluent solution. For radiolabelling, the reaction mixture was heated to 110 °C for 4 min and subsequently purified by passing the reaction mixture through a CM cartridge (Sep-Pak Accell Plus CM Plus Light Cartridge, Waters, Milford, USA). The drug substance [^68^Ga]Ga-DOTA was transferred through a sterile filter into the bulk vial and formulated with phosphate buffered saline. The quality control procedures included RP-HPLC, ITLC (colloid), pH, endotoxin and sterility testing, and ^68^Ge breakthrough measurement. The average yield was 900 MBq (*n* = 10 syntheses), and radiochemical purity was ≥ 95%.

### Dynamic [^68^Ga]Ga-DOTA PET acquisition and image reconstruction

PET/CT data were acquired on a silicon-photomultiplier-based Biograph Vision 600 PET/CT system (Siemens Healthineers, Erlangen, Germany). A scout and a low-dose CT scan were acquired for localization of the kidneys and for attenuation correction. Subsequently, dynamic PET scans were started simultaneously with [^68^Ga]Ga-DOTA injection; the mean applied activity was 112 MBq. Single-bed-position list-mode emission data were acquired for 30 min and image reconstruction was performed using 3-dimensional ordinary Poisson ordered-subsets expectation maximization with time-of-flight option. The data were resampled and reconstructed into frames of 24 × 5 s, 18 × 10 s, 10 × 30 s, and 20 × 60 s. Short time frames were chosen for the bolus phase directly after tracer injection to achieve a high temporal resolution of the arterial input function and renal cortical time-activity curves allowing to separate blood volume from blood flow effects.

### Analysis of dynamic renal PET images

The analysis consists of the estimation of the arterial input function and the construction of the renal cortical time-activity curves. The arterial input function was estimated from the PET signal in the abdominal aorta. The abdominal aorta (volume *V*_aorta_) was segmented in the CT images using a volume-of-interest (VOI) that was comprised of circular regions-of-interests (ROIs) of the aortal diameter on 15 continuous transversal slices. To compensate for partial volume effect and for robustness of arterial input function determination to patient motion, an oversized VOI-based method was applied [21–23]. An oversized VOI (volume *V*_oversized_) of 1.5 times the aortal diameter on each slice was drawn around the abdominal aorta (a VOI deemed to be large enough to include the entire aortal activity). To construct the background VOI (volume *V*_background_), several horse-shoe-shaped background ROIs were drawn outside of the oversized VOI. To subtract cross-contamination from surrounding background activity, the following equation was used to calculate the arterial input function (*C*: mean activity concentration in kBq/ml, *A*: total activity in kBq, *V*: volume in ml):

$$C_{\mathrm{aorta}}(\mathrm t)=\frac{A_{\mathrm{oversized}}\left(\mathrm t\right)-{(C}_{\mathrm{background}}\left(\mathrm t\right)\cdot{(V}_{\mathrm{oversized}}-V_{\mathrm{aorta}}))}{V_{\mathrm{aorta}}}$$

The plasma activity concentration (*C*_P_) was then calculated using the mean arterial activity concentration (*C*_aorta_) and the haematocrit value (HCT):

$${C_{\mathrm P}(t)=C}_{\mathrm{aorta}}(t)\cdot\frac1{(1-\mathrm{HCT})}$$

This simple conversion was previously described for the similar glomerularly filtered PET tracer [^68^Ga]Ga-NOTA in a pre-clinical study [14] and appears to be justified also for [^68^Ga]Ga-DOTA in humans due to its low plasma protein binding fraction of 2.8% ± 0.6% [16].

**Fig. 1:**
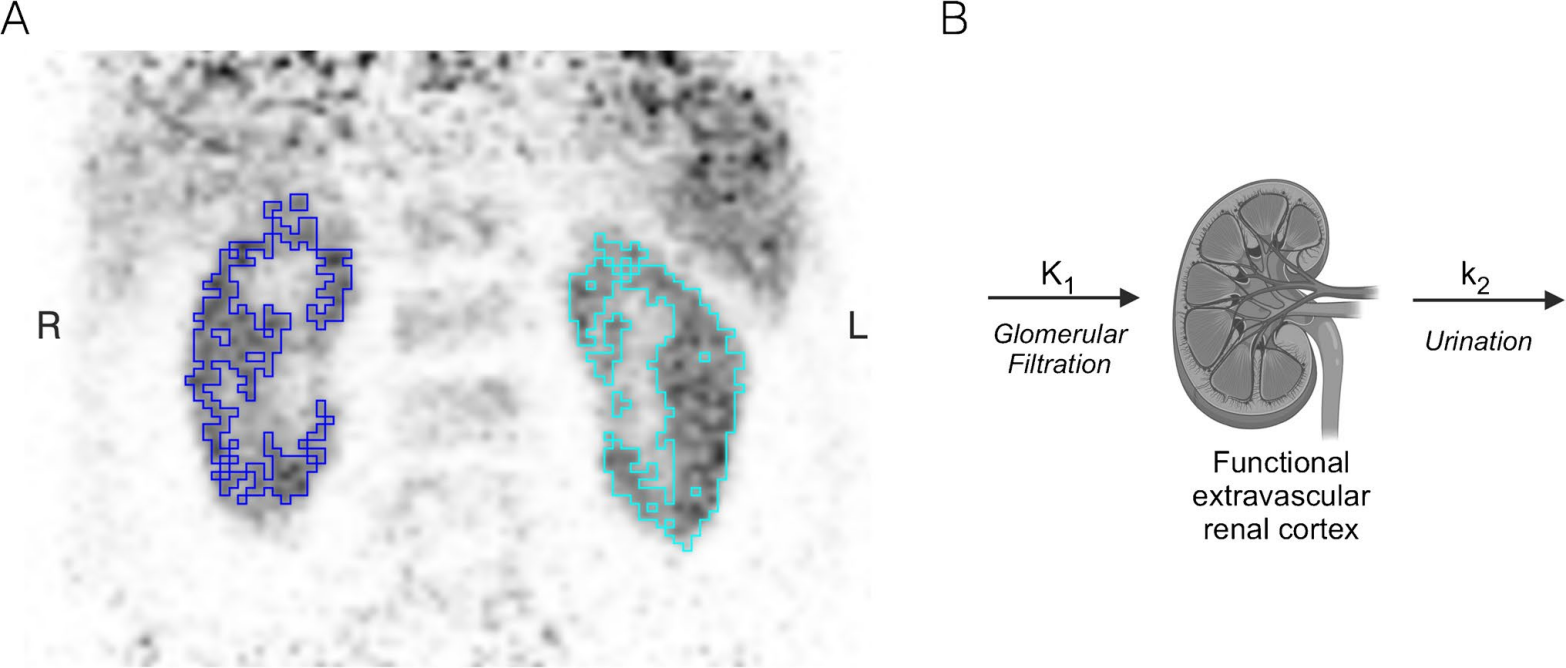
**A** Example of the segmentation of the functional renal cortex (frontal view of first PET frame). **B** Schematic representation of the single-compartmental tracer kinetic model

To construct the renal cortical time-activity curve, the functional renal cortex was segmented in the first PET frames that showed the influx of the tracer through the renal cortex (see Fig. 1A). The renal cortical VOI was drawn by application of a 3D auto iso-contour segmentation approach using hot pixel regional growing. The automatically-segmented VOIs were manually verified and adjusted to include the entire functional renal cortex. The volume of the respective VOI was defined as functional renal cortical volume *V*_RC_. Renal cortical time-activity curves (TACs) were created from the PET signal within *V*_RC_, separately for the left and for the right kidney.

Moreover, to evaluate activity in the urine excretion system, a 10-mm-diameter spherical VOI was evaluated in the left and in the right renal pelvis.

### Compartmental tracer kinetic modelling and GFR estimation

A 1-tissue compartmental model (Fig. 1B) was used to derive the GFR from dynamic PET data. A successful application of this model was previously described for a GFR derivation from [^68^Ga]Ga-NOTA PET data in rats [14]. We, therefore, choose the model as starting point for our evaluation of human dynamic [^68^Ga]Ga-DOTA PET data.

Assuming that [^68^Ga]Ga-DOTA enters the functional cortical renal volume exclusively by glomerular filtration and is not tubularly reabsorbed, the change of amount of activity in the  extravascular functional renal cortex (EC) can be described by the following differential equation according to Lee et al. [14]:

$$\frac{\mathrm dA_{\mathrm{EC}}}{\mathrm{dt}}=\mathrm{GFR}\cdot C_{\mathrm P}(\mathrm t)-{\mathrm k}_{\mathrm{urination}}\cdot A_{\mathrm{EC}}(\mathrm t)$$

Division by the extravascular functional renal cortical volume (*V*_EC_) yields:

$$\frac d{dt}\frac{A_{\mathrm{EC}}}{V_{\mathrm{EC}}}=\frac{\mathrm{GFR}}{V_{\mathrm{EC}}}\cdot C_{\mathrm P}\left(\mathrm t\right)-{\mathrm k}_{\mathrm{urination}}\cdot\frac{A_{\mathrm{EC}}\left(\mathrm t\right)}{V_{\mathrm{EC}}}$$

$$\Leftrightarrow\frac d{dt}C_{\mathrm{EC}}={\mathrm K}_1\cdot C_{\mathrm P}\left(\mathrm t\right)-{\mathrm k}_2\cdot C_{\mathrm{EC}}(\mathrm t)$$

with ${\mathrm{K}}_{1} = \mathrm{GFR} / \mathrm {V_{EC}}$, ${\mathrm{k}}_{2}= {\mathrm{k}}_{\mathrm{urination}}$, $V_{\mathrm{EC}}=V_{\mathrm{RC}}\cdot(1-\mathrm{vB})$, $\mathrm{vB}$: blood volume fraction, $(1 -\mathrm{ vB})$: extravascular volume fraction, and ${C}_{\mathrm{EC}}$: extravascular functional renal cortical activity concentration.

The solution to the differential equation is given by:

$$C_{EC}\left(t\right)=K_1\cdot C_P\left(t\right)\otimes e^{-k2t}=K_1\cdot\int\limits_o^tC_p\left(t\right)\cdot e^{-k_2\left(t-\tau\right)}d\tau$$

with the convolution integral ⊗.

The operational model curve that can be fitted to the renal cortical TACs is:

$$C_{\mathrm{model}}\left(\mathrm t\right)={(1-\mathrm{vB})\cdot C}_{\mathrm{EC}}\left(\mathrm t\right)+\mathrm{vB}\cdot C_{\mathrm{Aorta}}\left(\mathrm t\right)$$

with the fitting parameters ${\mathrm{K}}_{1}$, ${\mathrm{k}}_{2}$, and $\mathrm{vB}$. Compartmental kinetic modelling was separately performed for each kidney using the PKIN tool in PMOD.

The GFR was calculated as:

$${\mathrm{GFR}}_{\mathrm{total}}= {\mathrm{GFR}}_{\mathrm{left}} + {\mathrm{GFR}}_{\mathrm{right}}$$

$$=V_{\mathrm{RC},\mathrm{left}}\cdot\left(1-{\mathrm{vB}}_{\mathrm{left}}\right)\cdot{\mathrm K}_{1,\mathrm{left}}+V_{\mathrm{RC},\mathrm{right}}\cdot\left(1-{\mathrm{vB}}_{\mathrm{right}}\right)\cdot{\mathrm K}_{1,\mathrm{right}}$$

The PET-derived glomerular filtration rate was calculated from complete 30-min (GFR_PET-30_) and reduced 15-min (GFR_PET-15_) dynamic renal PET data sets to evaluate whether a shorter acquisition time is sufficient for an accurate estimation. Moreover, the PKIN tool allows to ignore data points (time intervals) in model fitting, which was used to exclude intervals with high urinary spill-over activity (see Results section). To derive the GFR from serum creatinine blood levels (GFR_CKD_), the established CKD-EPI equation [9] was used; the normalization to the body surface area was removed by multiplication with the body surface area.

### Analysis of renal scintigraphy images and renal time-activity curves

The clinically available [^99m^Tc]Tc-DTPA or [^99m^Tc]Tc-MAG3 renal scintigraphy that was acquired closest to the date of [^68^Ga]Ga-DOTA PET imaging was chosen. For 8/12 patients [^99m^Tc]Tc-DTPA data and for 4/12 [^99m^Tc]Tc-MAG3 data were available. Scintigraphy images were acquired in 36 frames of 10 s and 78 frames of 30 s on a Symbia S gamma camera (Siemens Healthineers, Erlangen, Germany) using a low energy high resolution collimator. Scans were started with DTPA/MAG3 injection (mean applied activity: 145 MBq of [^99m^Tc]Tc-DTPA and 71 MBq of [^99m^Tc]Tc-MAG3, respectively). To create renograms, C-shaped ROIs were drawn around each kidney [10]. Background correction was performed by subtraction of area-normalized background ROIs (C-shaped ROIs surrounding the lower, lateral, and upper part of the respective kidney). The renograms and PET-derived renal TACs were visually analysed in a consensus read by three nuclear medicine physicians (DK, MS, and CR). 

### Statistical analysis/software

All statistical evaluations were performed using R statistical software in version 4.0.3 (R Foundation for Statistical Computing, Vienna, Austria, www.R-project.org). The Pearson correlation coefficient (PCC) was determined to describe the correlation between different GFR estimates in a linear regression model. Additionally, the between-test correlation was evaluated using the intraclass correlation coefficient (ICC) in a two-way mixed effect model [24, 25]. The ICC is reported as ICC (lower confidence bound–upper confidence bound) as defined by Shrout and Fleiss [24]. According to the definition by Koo et al. [25], an ICC < 0.50 was regarded as poor, an ICC ≥ 0.50 and < 0.75 as moderate, an ICC ≥ 0.75 and < 0.90 as good, and an ICC ≥ 0.90 as excellent. The between-test agreement was evaluated in a Bland–Altman analysis [26].

Analysis of all PET/CT and scintigraphy images was performed using PMOD 4.202 (PMOD Technologies, Zurich, Switzerland); compartmental kinetic modelling was performed using the PKIN tool in PMOD. For data handling and visualization of renograms and renal TACs, MATLAB 2021b (MathWorks, Natick, USA) and OriginPro 2020b (OriginLab, Northampton, USA) were used. Graphics were created using BioRender.com (BioRender, San Francisco, USA, www.BioRender.com).

## Results

### Visual analysis of PET images and time-activity curves

**Fig. 2:**
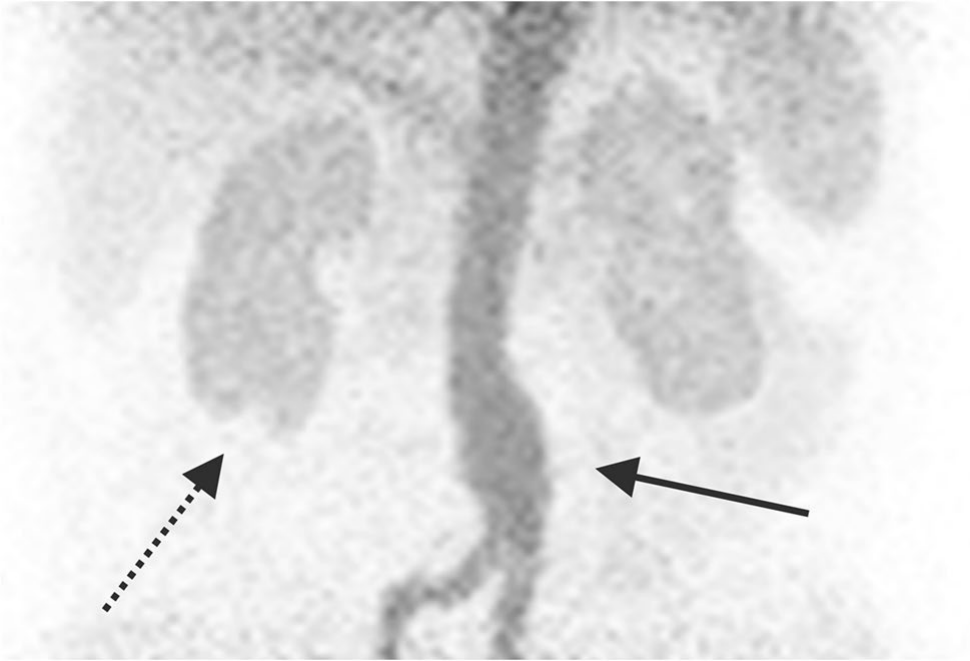
Maximum intensity projection (frontal view) of a left-sided abdominal aortic aneurysm (solid black arrow) and a cyst at the lower pole of the right kidney (dashed black arrow)

Image quality of dynamic [^68^Ga]Ga-DOTA PET images was higher than of planar scintigraphy images and showed additional anatomical details. For example, in one patient, a cyst at the lower pole of the right kidney was visualized (Fig. 2). Moreover, [^68^Ga]Ga-DOTA is a perfusion PET tracer that can visualize extrarenal vascular abnormalities. In the same patient, an aortic aneurysm was detected that had already been reported in previous CT examinations.

**Fig. 3:**
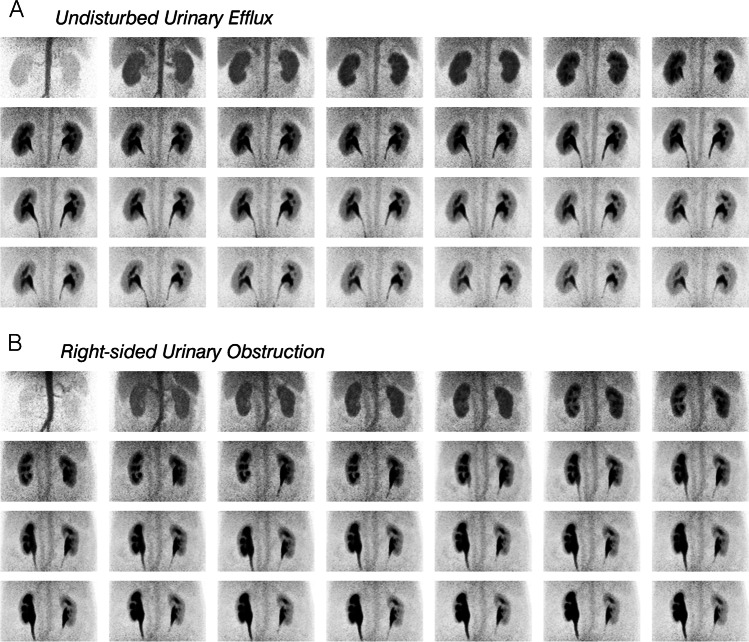
Maximum intensity projections (frontal view) of dynamic PET images (for visualization, PET data were resampled and reconstructed into 12 frames of 30 s and, thereafter, 16 frames of 90 s, from left to right). **A** Normal result (patient ID #8). **B** Pathological result (right-sided urinary obstruction, patient ID #12)

**Fig. 4:**
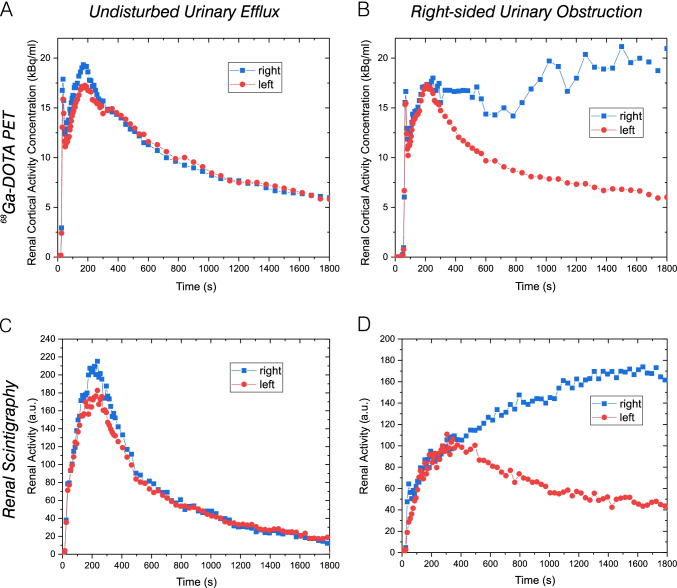
**A** and **B** PET-derived renal cortical TACs. **C** and **D** Scintigraphy-derived renograms. **A** and **C** Normal result (patient ID #8). **B** and **D** Pathological result (right-sided urinary obstruction, patient ID #12)

The visual analysis of dynamic [^68^Ga]Ga-DOTA PET images and renal cortical TACs revealed both-sided urinary obstruction in 2/12 patients, right-sided urinary obstruction in 1/12 patients, and no urinary obstruction in 9/12 patients. These were the same findings as in the renal scintigraphy examinations. Detailed results are given in Table 2. Figure 3 shows examples of normal (patient ID #8, Fig. 3A) and pathological (right-sided urinary obstruction, patient ID #12, Fig. 3B) dynamic renal PET images. PET renal TACs and scintigraphy renograms of the same patients are presented in Fig. 4.

**Table 2:** Visual interpretation results

Patient ID	Interval between scintigraphy and PET (d)	PET result	Scintigraphy result
1	− 57	No urinary obstruction	No urinary obstruction
2	− 113	No urinary obstruction	No urinary obstruction
3	− 3	No urinary obstruction	No urinary obstruction
4	46	No urinary obstruction	No urinary obstruction
5	40	No urinary obstruction	No urinary obstruction
6	84	No urinary obstruction	No urinary obstruction
7	21	No urinary obstruction	No urinary obstruction
8	85	No urinary obstruction	No urinary obstruction
9	29	Both-sided urinary obstruction	Both-sided urinary obstruction
10	− 71	Right-sided urinary obstruction	Right-sided urinary obstruction
11	126	No urinary obstruction	No urinary obstruction
12	15	Both-sided urinary obstruction	Both-sided urinary obstruction

*Results of the visual interpretation of dynamic [*^*68*^*Ga]Ga-DOTA PET data (images and TACs) and renal scintigraphy data (images and renograms)*

### Analysis of compartmental kinetic models

First, for all patients, GFR_PET-30_ (calculated from complete PET data sets) and GFR_PET-15_ (calculated from the first 15 min of the PET data sets) were calculated by compartmental kinetic modelling. The determined fit functions based on the 1-tissue compartment model were of limited quality and showed marked differences to the actual measurements. This was already noted by Lee et al. [14] for the similar tracer [^68^Ga]Ga-NOTA in rats and may show some limitations of this simple kinetic model, possibly caused by non-linear transfer of the tracer between compartments [14] or urine spill-over effects. We therefore expanded the model and implemented a dual spill-over correction (using the PET signal measured in the urine VOIs in the renal pelvises) to account for spill-over from activity in the urine tract. However, fit functions determined by the expanded model yielded poorer results than the original 1-tissue compartment model. A possible reason is a high variability in urine spill-over that cannot be represented in a simple model.

**Fig. 5:**
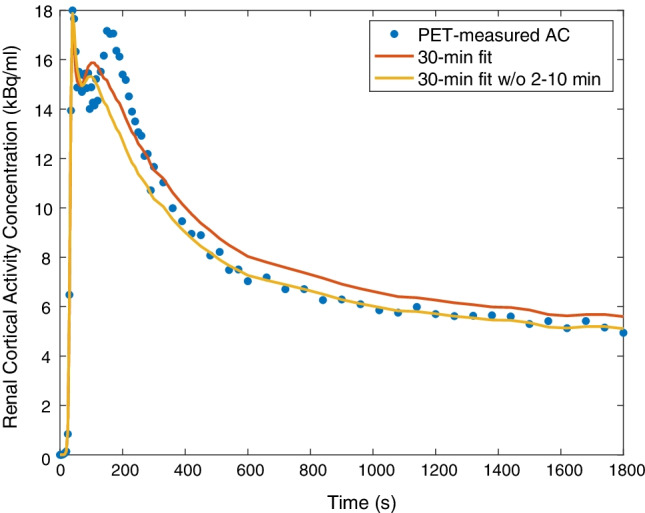
Exemplary presentation of model curves (patient ID #7, left kidney) showing the measured PET data and the determined fit functions based on the 1-tissue compartment model using the complete 30-min data set and the 30-min data set without minutes 2 to 10. AC: activity concentration

Analyses of the urine VOI time-activity curves revealed that the urine signal was delayed in comparison to the renal signal and most prominent in the first 2 to 10 min of the time-activity curves. We, therefore, decided to exclude this interval from the modelling approach to calculate GFR_PET-30_w/o2to10_ and GFR_PET-15_w/o2to10_, respectively. The fit functions obtained were of higher quality and showed a markedly increased agreement with the measured data after 10 min post-injection. In Fig. 5, exemplary model curves for the complete 30-min data set and the 30-min data set without minutes 2 to 10 are presented to demonstrate the effect on fit quality. Goodness-of-fit confirmed improved fit quality for the corrected model in terms of lower *χ*^2^ and AIC (Akaike information criterion) values. Detailed results for all modelling approaches and patients are given in Table 3.

**Table 3:** Goodness-of-fit kinetic modelling

Patient ID	*χ*^*2*^								Akaike information criterion							
	Left				Right				Left				Right			
	M1	M2	M3	M4	M1	M2	M3	M4	M1	M2	M3	M4	M1	M2	M3	M4
1	4.83	2.55	2.99	1.18	5.70	3.21	5.28	2.24	126.68	45.94	72.83	9.50	139.70	57.48	108.75	29.67
2	6.10	1.50	4.94	1.51	6.70	2.58	5.70	3.43	144.89	22.49	14.52	17.44	152.24	47.51	113.51	41.66
3	6.48	1.21	6.11	1.78	4.15	0.67	3.93	1.00	149.60	13.41	117.84	22.06	114.95	− 13.26	90.02	4.79
4	4.29	2.20	6.76	6.94	0.93	0.96	1.00	0.91	46.90	20.63	41.82	30.95	2.59	5.69	5.56	14.66
5	-	-	-	-	4.75	2.27	5.35	1.16	-	-	-	-	49.86	21.20	37.37	16.62
6	8.24	7.36	11.68	19.39	8.28	7.42	12.98	22.95	65.86	42.40	52.20	39.17	65.98	42.54	54.21	40.52
7	3.47	0.20	4.02	0.27	5.66	1.83	6.09	2.62	100.84	− 67.77	91.52	-34.96	139.08	31.99	117.74	33.65
8	4.32	1.40	2.57	1.11	6.28	1.62	4.66	1.91	118.38	19.89	63.38	7.71	147.19	26.46	100.82	24.13
9	2.73	2.46	2.39	2.99	4.40	2.73	3.35	3.07	68.12	38.96	50.81	35.33	98.71	42.90	69.08	36.13
10	2.32	2.83	2.62	3.77	2.95	3.06	3.17	4.23	62.93	44.35	61.63	41.84	79.60	47.29	73.16	45.04
11	2.65	0.42	3.14	0.48	2,43	0.36	2.78	0.44	79.97	− 33.77	75.98	− 17.16	73.07	− 41.19	68.37	− 20.24
12	8.90	0.55	8.06	0.55	10.06	8.12	1.09	1.27	174.42	− 22.40	135.35	− 13.40	183.93	99.06	9.58	11.95

χ^2^ and Akaike information criterion for the different kinetic modelling approaches; M1: complete 30-min data set (corresponding to GFR_PET-30_); M2: 30-min data set without minutes 2 to 10 (corresponding to GFR_PET-30_w/o2to10_); M3: reduced 15-min data set (corresponding to GFR_PET-15_); M4: 15-min data set without minutes 2 to 10 (corresponding to GFR_PET-15_w/o2to10_)

GFR_PET-30_, GFR_PET-30_w/o2to10_, GFR_PET-15_, GFR_PET-15_w/o2to10_, and GFR_CKD-EPI_ for all patients are presented in Table 4. The detailed kinetic modelling results are shown as Supplemental Material (Supplemental Table S1 for GFR_PET-30_, Supplemental Table S2 for GFR_PET-30_w/o2to10_, Supplemental Table S3 for GFR_PET-15_, and Supplemental Table S4 for GFR_PET-15_w/o2to10_).

**Table 4:** GFR results

Patient ID	GFR_CKD-EPI_ (ml/min)	GFR_PET-30_ (ml/min)	GFR_PET-30_w/o2to10_ (ml/min)	GFR_PET-15_ (ml/min)	GFR_PET-15_w/o2to10_ (ml/min)
1	80.6	72.9	71.0	68.8	68.9
2	59.4	46.9	43.7	45.2	43.3
3	90.9	82.0	76.6	79.3	75.0
4	92.2	89.1	73.7	89.4	72.0
5	89.8	84.1	63.7	88.4	64.9
6	73.8	85.5	63.7	88.4	74.3
7	87.9	84.0	88.6	83.0	87.9
8	132.8	128.9	107.6	122.8	104.9
9	103.1	56.2	62.3	61.1	62.8
10	67.9	41.3	49.0	41.4	49.2
11	98.8	91.7	79.6	90.7	79.9
12	85.9	70.3	52.0	74.4	66.6

*Serum creatinine and PET-derived GFR results*

### Comparison between GFR_PET__-30_/GFR_PET__-30_w/o2to10_ and GFR_CKD_

**Fig. 6:**
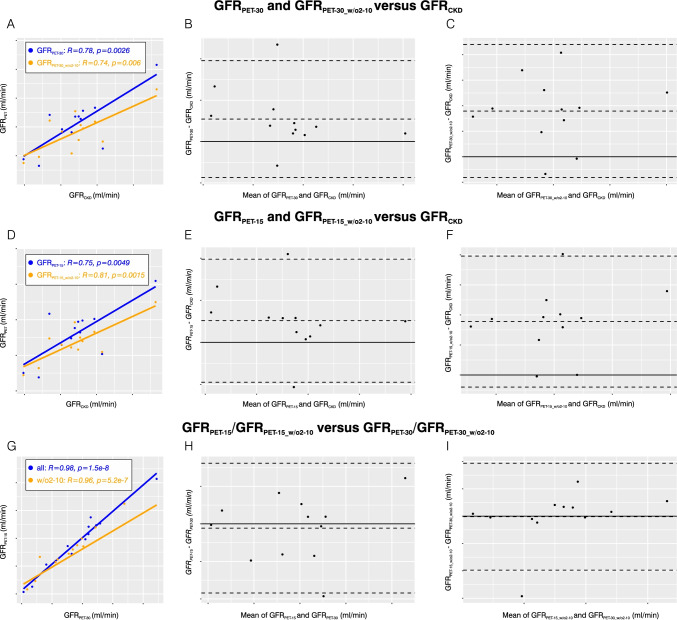
Correlation and agreement analyses for all patients (*n* = 12). **A** Scatter plot for GFR_PET-30_ and GFR_PET-30_w/o2to10_ versus GFR_CKD_. **B** Bland–Altman plot for GFR_PET-30_ versus GFR_CKD_. **C** Bland–Altman plot for GFR_PET-30_w/o2to10_ versus GFR_CKD_. **D** Scatter plot for GFR_PET-15_ and GFR_PET-15_w/o2to10_ versus GFR_CKD_. **E** Bland–Altman plot for GFR_PET-15_ versus GFR_CKD_. **F** Bland–Altman plot for GFR_PET-15_w/o2to10_ versus GFR_CKD_. **G** Scatter plot for GFR_PET-15_ versus GFR_PET-30_ and GFR_PET-15_w/o2to10_ versus GFR_PET-30_w/o2to10_. **H** Bland–Altman plot for GFR_PET-15_ versus GFR_PET-30_. **I** Bland–Altman plot for GFR_PET-15_w/o2to10_ versus GFR_PET-30_w/o2to10_

Regarding all patients, GFR_PET-30_ and GFR_CKD_ were well correlated with an ICC of 0.77 (lower bound–upper bound 0.46–0.91) and a PCC of 0.78 (95%-*CI* 0.38–0.94); Bland–Altman bias for GFR_PET-30_ was 10.86 (95%-*CI* 1.70–20.03) ml/min (Fig. 6 A and B). The optimized fit model yielded comparable results: GFR_PET-30_w/o2to10_ and GFR_CKD_ were correlated with an ICC of 0.74 (lower bound–upper bound 0.40–0.90) and a PCC of 0.74 (95%-*CI* 0.28–0.93), Bland–Altman bias for GFR_PET-30_w/o2to10_ was 17.90 (95%-*CI* 9.45–26.35) ml/min (Fig. 6 A and C).

**Fig. 7:**
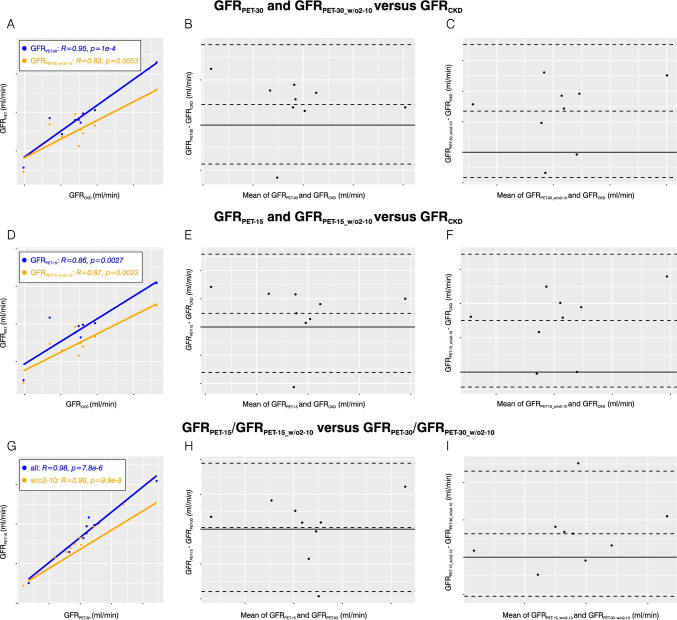
Correlation and agreement analyses for patients with undisturbed urinary efflux (*n* = 9). **A** Scatter plot for GFR_PET-30_ and GFR_PET-30_w/o2to10_ versus GFR_CKD_. **B** Bland–Altman plot for GFR_PET-30_ versus GFR_CKD_. **C** Bland–Altman plot for GFR_PET-30_w/o2to10_ versus GFR_CKD_. **D** Scatter plot for GFR_PET-15_ and GFR_PET-15_w/o2to10_ versus GFR_CKD_. **E** Bland–Altman plot for GFR_PET-15_ versus GFR_CKD_. **F** Bland–Altman plot for GFR_PET-15_w/o2to10_ versus GFR_CKD_. **G** Scatter plot for GFR_PET-15_ versus GFR_PET-30_ and GFR_PET-15_w/o2to10_ versus GFR_PET-30_w/o2to10_. **H** Bland–Altman plot for GFR_PET-15_ versus GFR_PET-30_. **I** Bland–Altman plot for GFR_PET-15_w/o2to10_ versus GFR_PET-30_w/o2to10_

Next, we separately analysed patients with undisturbed urinary efflux. In this subgroup (*n* = 9), GFR_PET-30_ and GFR_CKD_ were excellently correlated with an ICC of 0.95 (0.83–0.98) and a PCC of 0.95 (95%-*CI*: 0.77–0.99). The agreement between GFR_PET-30_ and GFR_CKD_ was high with a Bland–Altman bias for GFR_PET-30_ of 4.59 (− 0.61–9.78) ml/min (Fig. 7A and B). Correlations for the optimized fit model were slightly deteriorated: GFR_PET-30_w/o2to10_ and GFR_CKD_ were correlated with an ICC of 0.82 (0.50–0.95) and a PCC of 0.83 (95%-*CI*: 0.37–0.97), that is, still a good correlation was found. Bland–Altman bias for GFR_PET-30_w/o2to10_ was 13.47 (4.93–22.00) ml/min (Fig. 7A and C).

### Comparison between GFR_PET__-15_/GFR_PET__-15_w/o2to10_ and GFR_CKD_

Regarding all patients, GFR_PET-15_ and GFR_CKD_ were moderately correlated with an ICC of 0.74 (0.40–0.90) and a PCC of 0.75 (0.30–0.93); Bland–Altman bias for GFR_PET-15_ was 10.30 (0.81–19.79) ml/min (Fig. 6D and E). The optimized fit model yielded improved results: GFR_PET-15_w/o2to10_ and GFR_CKD_ were well correlated with an ICC of 0.80 (0.52–0.92) and a PCC of 0.81 (0.43–0.95), Bland–Altman bias for GFR_PET-15_w/o2to10_ was 17.94 (10.72–24.86) ml/min (Fig. 6D and F).

In the subgroup of patients with undisturbed urinary efflux (*n* = 9), the PCC between GFR_PET-15_ and GFR_CKD_ was 0.86 (0.46–0.98). GFR_PET-15_ and GFR_CKD_ were well correlated with an ICC of 0.86 (0.60–0.96). The agreement between GFR_PET-15_ and GFR_CKD_ was high with a Bland–Altman bias for GFR_PET-15_ of 4.84 (− 3.40–13.08) ml/min (Fig. 7D and E). Correlations for the optimized fit model were slightly improved: GFR_PET-15_w/o2to10_ and GFR_CKD_ were well correlated with an ICC of 0.86 (0.57–0.96) and a PCC of 0.87 (0.48–0.98), Bland–Altman bias for GFR_PET-15_w/o2to10_ was 15.04 (7.43–9.90) ml/min (Fig. 7D and F).

### Comparison between GFR_PET__-15_/GFR_PET__-15_w/o2to10_ and GFR_PET__-30_/GFR_PET__-30_w/o2to10_

The comparison between GFR_PET-15_ and GFR_PET-30_ revealed an excellent correlation with an ICC of 0.98 (0.95–0.99) and a PCC of 0.98 (0.93–1.0). There was a high agreement between GFR_PET-15_ and GFR_PET-15_ with a Bland–Altman bias for GFR_PET-15_ of − 0.57 (− 3.38–2.24) ml/min (Fig. 6G and H). Likewise, the comparison between GFR_PET-15_w/o2to10_ and GFR_PET-30_w/o2to10_ revealed an excellent correlation with an ICC of 0.96 (0.89–0.99) and a PCC of 0.96 (0.87–0.99). There was a high agreement between GFR_PET-15_w/o2to10_ and GFR_PET-30_w/o2to10_ with a Bland–Altman bias for GFR_PET-15_w/o2to10_ of − 0.10 (− 3.28–3.07) ml/min (Fig. 6G and I).

Also, for the subgroup of patients with undisturbed urinary efflux (*n* = 9), an excellent correlation was observed between GFR_PET-15_ and GFR_PET-30_ indicated by an ICC of 0.98 (0.93–0.99) and a PCC of 0.98 (0.88–1.00). Bland–Altman bias for GFR_PET-15_ was 0.25 (− 3.36–3.87) ml/min (Fig. 7G and H). Likewise, the comparison between GFR_PET-15_w/o2to10_ and GFR_PET-30_w/o2to10_ revealed an excellent correlation with an ICC of 0.99 (0.97–1.00) and a PCC of 0.99 (0.96–1.00). There was a high agreement between GFR_PET-15_w/o2to10_ and GFR_PET-30_w/o2to10_ with a Bland–Altman bias for GFR_PET-15_w/o2to10_ of 1.57 (− 0.08–3.22) ml/min (Fig. 7G and I).

## Discussion

Several PET tracers have been proposed for renal imaging in humans including the GFR tracers [^68^Ga]Ga-EDTA, [^68^Ga]Ga-NOTA, and [^68^Ga]Ga-DOTA [13–16] and renal perfusion tracers like ^82^Rb and [^15^O]H_2_O [27, 28]. Possible advantages of renal PET imaging over conventional scintigraphy imaging are an improved image quality, resolution, and contrast [27]. This can allow a more precise delineation of renal parenchyma, blood vessels, and background [29]. Particularly, the evaluation of patients with complex anatomy might benefit from 3D PET imaging [13]. Moreover, in times of supply shortages of ^99^Mo/^99m^Tc-generators [30], renal PET ^68^Ga-based tracers may be an appropriate alternative to renal scintigraphy and may allow optimized utilization of cost-intensive ^68^Ge/^68^Ga-generators.

In this study, visual interpretation of dynamic [^68^Ga]Ga-DOTA PET images and renal cortical TACs in radionuclide therapy patients revealed the same results as conventional scintigraphy indicating that [^68^Ga]Ga-DOTA PET is a suitable alternative. Image quality of renal PET was higher than of renal scintigraphy images. Additionally, the examination of renal parenchyma in high resolution PET images allowed for assessment of kidney morphology. In one patient, a kidney cyst was detected (Fig. 2). To evaluate a possible benefit in clinical routine, a systematic comparison with [^99m^Tc]Tc-DMSA SPECT imaging as a gold standard for examination of renal parenchyma would be desirable in future studies.

Glomerularly filtered PET tracers allow an estimation of the GFR by different methods. Hofman et al. [15] reported that a GFR estimation by repeated [^68^Ga]Ga-EDTA plasma sampling (comparable to [^51^Cr]Cr-EDTA plasma sampling) is feasible. Moreover, a good correlation between the PET-derived rate of excretion into bladder, ureters, and kidneys (measured in 10-min dynamic PET scans) and the plasma sampling-derived GFR was observed [15]. However, using this approach, the GFR cannot directly be calculated, but is indirectly derived from a correlation with the plasma sampling-derived values which can themselves be erroneous.

An alternative is to directly calculate the GFR without laborious and invasive repeated blood sampling from dynamic PET data by compartmental tracer kinetic modelling of glomerularly filtered PET tracers. Lee et al. [14] described the feasibility of a GFR calculation by single-compartmental tracer kinetic analysis from [^18^F]fluoride and [^68^Ga]-NOTA PET data in rats. In this study, we report the, to our knowledge, first investigation of a human GFR estimation by compartmental tracer kinetic modelling of dynamic PET data.

The determined fit functions based on a simple 1-tissue compartment model introduced for preclinical PET imaging were of limited quality and showed differences to the actual measurements. This was also reported in the preclinical study for [^68^Ga]Ga-NOTA [14]. A possible reason is spill-over from urinary radioactivity levels. An implementation of a dual spill-over correction to our model for urine activity did not yield satisfying results. An explanation might be a complex variability of urine spill-over that cannot be represented in a linear kinetic model. As an analysis of the urine time-activity-curves showed a major contribution of urine activity in the minutes 2 to 10 after tracer injection (delayed to the maximum of renal time-activity curves), we excluded this interval from kinetic modelling and yielded substantial improvements in fit quality of modelled time-activity curves (Fig. 5 and Table 3).

Regarding all patients, PET-based GFR_PET_ estimations showed a high correlation to the serum creatinine-derived GFR_CKD_ values that are commonly used in clinical routine practice (Fig. 6). Interestingly, for complete 30-min data sets, correlation was not improved for the modified model excluding spill-over biased data. However, if only the first 15 min of dynamic PET data were included, the correlation was higher for the modified model. A possible explanation is that GFR_CKD_, which was used as reference standard in this study, itself is prone to errors and, therefore, cannot be regarded as universal gold standard [7]. However, defining an improved reference standard is difficult, as all available methods are restricted by specific limitations. A direct absolute GFR measurement is ethically not justified. The clinically-established GFR measurement by urine creatinine clearance is limited due to frequent urine collection errors [2]. Nuclear medicine examination techniques like [^99m^Tc]Tc-DTPA scintigraphy would require multiple tracer injections in short temporal distance for a direct comparison. Most likely, a comparison against the clinical gold standard of a GFR derivation by inulin clearance could be used to validate the accuracy of GFR_PET_ in future studies, but the procedure is laborious, invasive, and not established in clinical routine practice.

If patients with urinary obstruction were excluded, the correlations of GFR_PET_ to GFR_CKD_ were increased; for the evaluation of complete 30-min data set, in this group, an excellent correlation was found (Fig. 7). In patients with urinary obstruction, the shape of the time-activity curves with plateau formation might lead to an impeded description by the applied mathematical model, as a urinary efflux might insufficiently be described by a linear model with a kinetic constant k_2_. Consequently, the assumption of a linear differential equation for the temporal change of activity in the renal cortex might lead to deviations due to the time-dependent urinary reflux. More complex kinetic models might be necessary for a more accurate GFR calculation in patients with urinary obstruction; these may involve additional compartments, higher-order transfer between compartments, or an explicit spill-over correction of urinary radioactivity levels. Future studies including more patients with urinary obstruction are required for a detailed investigation and establishment of an optimized kinetic model.

A good agreement was demonstrated between GFR_PET-30_ (derived from complete 30-min PET data sets) and GFR_PET-15_ (derived from reduced 15-min PET data sets) for both the standard model and the modified model to exclude urine spill-over. Therefore, an evaluation of urinary efflux and a PET-based GFR examination within 15 min examination time appears feasible by dynamic [ ^68^Ga]Ga-DOTA PET. Short examination times increase patient comfort and can decrease contact time and thus the risk of infection in the ongoing COVID-19 pandemic.

Possible scenarios for an application of PET-derived GFR measurements could include monitoring in pharmacological trials, as repeated measurements might allow for quasi-real time assessment of kidney function in patients/probands with unimpaired kidney function. Prior to a broader implementation of the technique, larger studies should be performed to validate the results of our first experiences. These could also include a repeatability analysis to assess the reliability of the technique.

The study faces several limitations. First, the number of patients was low and included patients presented concomitant malignant comorbidities but no chronic kidney diseases. Particularly, an investigation of patients with low GFR values would be of additional interest to validate the method for patients with decreased renal function. Next, PET data were compared to mixed [^99m^Tc]Tc-DTPA and [^99m^Tc]Tc-MAG3 renal scintigraphy results which were acquired in variable temporal distance to the PET scans. However, an influence on the assessment of urinary extraction is unlikely and GFR_PET_ results were compared to GFR_CKD_ results derived from creatinine serum levels which were taken on the day of the PET scan. Finally, we noticed that respiratory motion had some influence on the location of the kidneys in different time frames, thus potentially contributing to the limited quality of compartment model fits. Respiratory gating/motion correction may minimize this effect in future investigation.

Potential improvements for future approaches of PET-derived GFR measurements may include an evaluation of the other glomerularly filtered PET-tracers [^68^Ga]Ga-EDTA and [^68^Ga]Ga-NOTA. These exhibit a lower protein binding fraction (0.1 ± 0.0% for [^68^Ga]Ga-NOTA and 1.2 ± 0.6% for [^68^Ga]Ga-EDTA versus 2.8 ± 0.6% for [^68^Ga]Ga-DOTA after 10 min in human serum), which could lead to higher accuracy of kinetic modelling [16]. Moreover, a high accuracy of a GFR derived by kinetic modelling of PET data for the tubularly-secreted PET tracer [^18^F]fluoride was reported in rats [14]. If an exclusive excretion by glomerular filtration is no prerequisite for PET-derived GFR measurements, an evaluation of the feasibility of GFR estimations from dynamic PET data using DOTATOC/DOTATATE and PSMA tracers might be of clinical interest, as tracer uptake and kidney function could be evaluated in a single PET examination prior to radionuclide therapy.

## Conclusion

Visual interpretation of dynamic PET images and renal TACs revealed comparable results to conventional scintigraphy renograms indicating that [^68^Ga]Ga-DOTA PET can be a suitable alternative. The non-invasive GFR estimation by single-compartmental-modelling of dynamic [^68^Ga]Ga-DOTA PET data is feasible and shows a good correlation to serum creatinine-derived GFR values. In patients with undisturbed urinary efflux, the correlation was excellent. Dynamic PET data acquisition for 15 min is sufficient for visual evaluation and GFR derivation.

## Supplementary Information

Below is the link to the electronic supplementary material.

Supplementary file1 (DOCX 33 KB)

## Data Availability

The datasets generated and/or analysed during the current study are not publicly available due to privacy legislation but are available from the corresponding author on reasonable request.
