# Supplementary material for: First experiences with dynamic renal [68Ga]Ga-DOTA PET/CT: a comparison to renal scintigraphy and compartmental modelling to non-invasively estimate the glomerular filtration rate
Source: Eur J Nucl Med Mol Imaging. 2022 Apr 12;49(10):3373–86. doi: 10.1007/s00259-022-05781-1 (PMC9002049; doi:10.1007/s00259-022-05781-1)
Supplement: Supplementary file 1 — Supplementary file1 (DOCX 33 KB) [file 259_2022_5781_MOESM1_ESM.docx]

### SUPPLEMENTAL MATERIAL

#### SUPPLEMENTAL TABLE S1

#### Kinetic Modelling Results Complete 30-min data set (corresponding to GFR_PET-30_)

| **Pat.**  **ID** | **V_RC left_**  **(cm^3^)** | **V_RC right_**  **(cm^3^)** | **K1_left_**  **(ml/cm^3^/min)** | **K1_right_**  **(ml/cm^3^/min)** | **V_B left_**  **(ml)** | **V_B right_**  **(ml)** | **k2_left_**  **(min^-1^)** | **k2_right_**  **(min^-1^)** | **GFR_left_**  **(ml/min)** | **GFR_right_**  **(ml/min)** | **GFR_total_**  **(ml/min)** |
| --- | --- | --- | --- | --- | --- | --- | --- | --- | --- | --- | --- |
| 1 | 57.7 | 64.6 | 0.80 | 0.72 | 0.21 | 0.22 | 0.40 | 0.20 | 36.8 | 36.2 | 72.9 |
| 2 | 83.0 | 77.4 | 0.32 | 0.33 | 0.09 | 0.11 | 0.45 | 0.44 | 24.1 | 22.9 | 46.9 |
| 3 | 61.1 | 67.3 | 0.85 | 0.81 | 0.23 | 0.23 | 0.73 | 0.60 | 40.1 | 41.9 | 82.0 |
| 4 | 103.0 | 127.0 | 0.84 | 0.44 | 0.35 | 0.41 | 0.85 | 0.32 | 56.3 | 32.7 | 89.1 |
| 5 | 0 | 110.0 | - | 0.82 | - | 0.07 | - | 0.77 | 0 | 84.1 | 84.1 |
| 6 | 121.0 | 85.0 | 0.50 | 0.75 | 0.33 | 0.29 | 0.62 | 0.88 | 40.0 | 45.5 | 85.5 |
| 7 | 56.6 | 70.1 | 0.69 | 0.74 | 0.07 | 0.08 | 0.95 | 0.84 | 36.4 | 47.6 | 84.0 |
| 8 | 138.4 | 77.8 | 0.68 | 0.82 | 0.17 | 0.20 | 0.46 | 0.54 | 77.8 | 51.0 | 128.9 |
| 9 | 70.8 | 86.1 | 0.47 | 0.43 | 0.21 | 0.19 | 0.40 | 0.35 | 26.5 | 29.8 | 56.2 |
| 10 | 101.4 | 50.4 | 0.31 | 0.36 | 0.16 | 0.18 | 0.28 | 0.29 | 26.5 | 14.8 | 41.3 |
| 11 | 92.6 | 91.2 | 0.58 | 0.57 | 0.13 | 0.14 | 0.55 | 0.52 | 46.9 | 44.8 | 91.7 |
| 12 | 128.7 | 78.3 | 0.41 | 0.29 | 0.06 | 0.09 | 0.44 | 0.15 | 49.6 | 20.7 | 70.3 |

*Supplemental Table S1: Kinetic modelling results for the complete 30-min dynamic ^68^Ga-DOTA renal PET/CT data sets.*

#### SUPPLEMENTAL TABLE S2

#### Kinetic Modelling Results 30-min data set without minutes 2 to 10 (corresponding to GFR_PET-30_w/o2to10_)

| **Pat.**  **ID** | **V_RC left_**  **(cm^3^)** | **V_RC right_**  **(cm^3^)** | **K1_left_**  **(ml/cm^3^/min)** | **K1_right_**  **(ml/cm^3^/min)** | **V_B left_**  **(ml)** | **V_B right_**  **(ml)** | **k2_left_**  **(min^-1^)** | **k2_right_**  **(min^-1^)** | **GFR_left_**  **(ml/min)** | **GFR_right_**  **(ml/min)** | **GFR_total_**  **(ml/min)** |
| --- | --- | --- | --- | --- | --- | --- | --- | --- | --- | --- | --- |
| 1 | 57.7 | 64.6 | 0.76 | 0.75 | 0.23 | 0.23 | 0.42 | 0.22 | 33.7 | 37.3 | 71.0 |
| 2 | 83.0 | 77.4 | 0.30 | 0.31 | 0.10 | 0.12 | 0.51 | 0.49 | 22.7 | 21.0 | 43.7 |
| 3 | 61.1 | 67.3 | 0.83 | 0.75 | 0.24 | 0.25 | 0.90 | 0.66 | 38.7 | 37.9 | 76.6 |
| 4 | 103.0 | 127.0 | 0.74 | 0.37 | 0.38 | 0.44 | 0.82 | 0.27 | 47.0 | 26.7 | 73.7 |
| 5 | 0 | 110.0 | - | 0.67 | - | 0.13 | - | 0.66 | - | 63.7 | 63.7 |
| 6 | 121.0 | 85.0 | 0.49 | 0.69 | 0.31 | 0.32 | 0.64 | 0.89 | 40.4 | 40.2 | 80.5 |
| 7 | 56.6 | 70.1 | 0.76 | 0.75 | 0.06 | 0.08 | 1.14 | 0.97 | 40.4 | 48.3 | 88.6 |
| 8 | 138.4 | 77.8 | 0.59 | 0.72 | 0.20 | 0.23 | 0.46 | 0.57 | 64.7 | 42.9 | 107.6 |
| 9 | 70.8 | 86.1 | 0.50 | 0.48 | 0.21 | 0.18 | 0.40 | 0.36 | 28.2 | 34.1 | 62.3 |
| 10 | 101.4 | 50.4 | 0.37 | 0.40 | 0.15 | 0.17 | 0.33 | 0.34 | 32.4 | 16.6 | 49.0 |
| 11 | 92.6 | 91.2 | 0.53 | 0.50 | 0.15 | 0.16 | 0.53 | 0.49 | 41.4 | 38.3 | 79.6 |
| 12 | 128.7 | 78.3 | 0.35 | 0.16 | 0.07 | 0.11 | 0.44 | 0.06 | 41.2 | 10.9 | 52.0 |

*Supplemental Table S2: Kinetic modelling results for the 30-min dynamic ^68^Ga-DOTA renal PET/CT data sets without minutes 2 to 10.*

#### SUPPLEMENTAL TABLE S3

#### Kinetic Modelling Results Reduced 15-min data set (corresponding to GFR_PET-15_)

| **Pat.**  **ID** | **V_RC left_**  **(cm^3^)** | **V_RC right_**  **(cm^3^)** | **K1_left_**  **(ml/cm^3^/min)** | **K1_right_**  **(ml/cm^3^/min)** | **V_B left_**  **(ml)** | **V_B right_**  **(ml)** | **k2_left_**  **(min^-1^)** | **k2_right_**  **(min^-1^)** | **GFR_left_**  **(ml/min)** | **GFR_right_**  **(ml/min)** | **GFR_total_**  **(ml/min)** |
| --- | --- | --- | --- | --- | --- | --- | --- | --- | --- | --- | --- |
| 1 | 57.7 | 64.6 | 0.77 | 0.69 | 0.22 | 0.24 | 0.36 | 0.17 | 34.9 | 33.9 | 68.8 |
| 2 | 83.0 | 77.4 | 0.31 | 0.32 | 0.10 | 0.11 | 0.41 | 0.40 | 23.1 | 22.0 | 45.2 |
| 3 | 61.1 | 67.3 | 0.83 | 0.79 | 0.23 | 0.24 | 0.68 | 0.56 | 38.8 | 40.5 | 79.3 |
| 4 | 103.0 | 127.0 | 0.84 | 0.44 | 0.35 | 0.42 | 0.84 | 0.32 | 56.7 | 32.7 | 89.4 |
| 5 | 0 | 110.0 | - | 0.85 | - | 0.05 | - | 0.79 | - | 88.4 | 88.4 |
| 6 | 121.0 | 85.0 | 0.65 | 0.69 | 0.27 | 0.36 | 0.80 | 0.81 | 57.4 | 37.7 | 95.1 |
| 7 | 56.6 | 70.1 | 0.69 | 0.73 | 0.07 | 0.08 | 0.94 | 0.81 | 36.1 | 46.9 | 83.0 |
| 8 | 138.4 | 77.8 | 0.65 | 0.79 | 0.18 | 0.20 | 0.41 | 0.49 | 73.9 | 48.9 | 122.8 |
| 9 | 70.8 | 86.1 | 0.50 | 0.47 | 0.21 | 0.18 | 0.45 | 0.43 | 28.0 | 33.1 | 61.1 |
| 10 | 101.4 | 50.4 | 0.32 | 0.35 | 0.16 | 0.18 | 0.29 | 0.28 | 27.0 | 14.4 | 41.4 |
| 11 | 92.6 | 91.2 | 0.58 | 0.56 | 0.13 | 0.14 | 0.54 | 0.51 | 46.5 | 44.2 | 90.7 |
| 12 | 128.7 | 78.3 | 0.40 | 0.36 | 0.06 | 0.08 | 0.40 | 0.26 | 48.1 | 26.3 | 74.4 |

*Supplemental Table S3: Kinetic modelling results for the reduced 15-min dynamic ^68^Ga-DOTA renal PET/CT data sets.*

#### SUPPLEMENTAL TABLE S4

#### Kinetic Modelling Results 15-min data set without minutes 2 to 10 (corresponding to GFR_PET-15_w/o2to10_).

| **Pat.**  **ID** | **V_RC left_**  **(cm^3^)** | **V_RC right_**  **(cm^3^)** | **K1_left_**  **(ml/cm^3^/min)** | **K1_right_**  **(ml/cm^3^/min)** | **V_B left_**  **(ml)** | **V_B right_**  **(ml)** | **k2_left_**  **(min^-1^)** | **k2_right_**  **(min^-1^)** | **GFR_left_**  **(ml/min)** | **GFR_right_**  **(ml/min)** | **GFR_total_**  **(ml/min)** |
| --- | --- | --- | --- | --- | --- | --- | --- | --- | --- | --- | --- |
| 1 | 57.7 | 64.6 | 0.74 | 0.73 | 0.23 | 0.23 | 0.35 | 0.19 | 32.9 | 36.0 | 68.9 |
| 2 | 83.0 | 77.4 | 0.30 | 0.31 | 0.10 | 0.12 | 0.47 | 0.45 | 22.5 | 20.8 | 43.3 |
| 3 | 61.1 | 67.3 | 0.81 | 0.75 | 0.24 | 0.26 | 0.84 | 0.64 | 37.5 | 37.5 | 75.0 |
| 4 | 103.0 | 127.0 | 0.74 | 0.37 | 0.40 | 0.44 | 0.85 | 0.26 | 45.7 | 26.3 | 72.0 |
| 5 | 0 | 110.0 | - | 0.68 | - | 0.13 | - | 0.64 | - | 64.9 | 64.9 |
| 6 | 121.0 | 85.0 | 0.47 | 0.64 | 0.32 | 0.35 | 0.66 | 0.97 | 38.5 | 35.7 | 74.2 |
| 7 | 56.6 | 70.1 | 0.76 | 0.74 | 0.06 | 0.09 | 1.16 | 0.92 | 40.6 | 47.2 | 87.9 |
| 8 | 138.4 | 77.8 | 0.57 | 0.70 | 0.21 | 0.23 | 0.41 | 0.52 | 63.1 | 41.8 | 104.9 |
| 9 | 70.8 | 86.1 | 0.51 | 048 | 0.21 | 0.18 | 0.43 | 0.38 | 28.6 | 34.3 | 62.8 |
| 10 | 101.4 | 50.4 | 0.38 | 0.40 | 0.14 | 0.17 | 0.35 | 0.34 | 32.7 | 16.6 | 49.3 |
| 11 | 92.6 | 91.2 | 0.53 | 0.50 | 0.15 | 0.17 | 0.55 | 0.48 | 41.7 | 38.1 | 79.9 |
| 12 | 128.7 | 78.3 | 0.34 | 0.35 | 0.07 | 0.08 | 0.43 | 0.25 | 41.0 | 25.6 | 66.6 |

*Supplemental Table S4: Kinetic modelling results for the 15-min dynamic ^68^Ga-DOTA renal PET/CT data sets without minutes 2 to 10.*
